# Scalable cyberinfrastructure for experimental NMR data

**DOI:** 10.1038/s41597-025-06446-y

**Published:** 2025-12-17

**Authors:** Jeffrey C. Hoch, Katherine Henzler-Wildman, Arthur S. Edison, Chad M. Rienstra, Christopher Bontempi, Jonathan R. Wedell, Gerard Weatherby, Harrison Burr, Yulia Pustovalova, Seenat Thongdee, Michael R. Gryk, Alexandra Pozhidaeva, Bernd Simon, Qi Cheng, Michael P. Wilson, Ion I. Moraru, Laura Morris, John N. Glushka, Mario Uchimiya, Alexander Eletsky, Abigail E. Moore, John H. Grimes, Alexander L. Paterson, Songlin Wang, Paulo R. Pinheiro, Boden H. Vanderloop, Mark W. Maciejewski

**Affiliations:** 1 Department of Molecular Biology & Biophysics, UConn Health, Farmington, CT 06030 USA; 2 Gregory P. Mullen NMR Structural Biology Facility, UConn Health, Farmington, CT 06030 USA; 3 Department of Biochemistry, University of Wisconsin–Madison, Madison, WI 53706 USA; 4 National Magnetic Resonance Facility at Madison (NMRFAM), University of Wisconsin–Madison, Madison, Wisconsin 53706 USA; 5 Department of Biochemistry & Molecular Biology, University of Georgia, Athens, Georgia 30602 USA; 6 Complex Carbohydrate Research Center (CCRC), University of Georgia, Athens, Georgia 30602 USA; 7 Institute for Bioinformatics, University of Georgia, Athens, Georgia 30602 USA; 8 The Richard D. Berlin Center for Cell Analysis & Modeling, UConn Health, Farmington, CT 06030 USA

**Keywords:** Research data, NMR spectroscopy

## Abstract

The Network for Advanced NMR (NAN) is a novel distributed resource that connects Nuclear Magnetic Resonance (NMR) facilities via a scalable cyberinfrastructure supporting NMR data harvesting, interactive data management, and the discovery of instruments, methods, and data to enable emerging data standards in biomedicine, chemistry, and material science. Anchored by the first open-access 1.1 GHz instruments in the USA, NAN integrates NMR facilities around a centralized hub for identity management, resource discovery, and access control. The system includes automated data harvesting through the NAN data transport system (NDTS), metadata-rich data archiving, and interactive web-based tools for data and metadata browsing, editing, and publishing, as well as tools for facility and laboratory data management by facility managers and principal investigators. NAN knowledgebases provide vetted, standardized pulse programs, protocols, parameters, and example datasets, along with processed data. Supported by the US National Science Foundation Midscale Research Infrastructure program, NAN helps to democratize access to NMR resources and fosters open, reproducible science.

## Introduction

Nuclear magnetic resonance (NMR) spectroscopy is one of the most versatile analytic methods for investigating the composition, structure, and dynamics of matter. Non-destructive and non-invasive, NMR can identify and quantify constituents of complex mixtures, report on the extent and timescale of fluctuations in both ordered and disordered systems, detect and quantify weak binding interactions in solution, and determine the three-dimensional structure of biological macromolecules. It plays an important role in chemistry and material science fields, probing amorphous and disordered materials, surfaces and other non-periodic environments, and chemical dynamics over timescales from nanoseconds to days. Applicable to both liquid and solid state, NMR has been described as an “evergreen”^1^ because it readily adapts to new applications.

NMR, as currently practiced at the challenging frontiers, is not for the faint of heart. High sensitivity and resolution demand ultra-high magnetic fields, requiring expensive superconducting magnets that are difficult to site and expensive to operate^2^. The versatility of NMR is also an Achilles Heel, because the number and variety of available experimental protocols pose a challenging barrier to entry. Becoming an expert in NMR typically involves a long apprenticeship in one of the “cathedrals” of NMR equipped to host this complex instrumentation.

Analysis of NMR data is equally if not more daunting. NMR spectra are information rich but make interpretation more challenging. The complexity of NMR spectra for large systems and mixtures frequently requires months of analysis for interpretation, with complex computational workflows and manual intervention at many stages^3–5^. The complexity of NMR applications to material science and chemistry are frequently lower, but they face their own challenges of sensitivity and resolution. Reproducing or validating NMR experiments and analyses is hindered by the lack of avenues for sharing empirical NMR data. Large collections of experimental data suitable for applications of recent advances in machine learning remain a distant objective. The need for expensive instrumentation, coupled with the high activation barrier NMR novices face when attempting to apply leading-edge experiments to their research questions, presents serious challenges for the average researcher who may benefit from the use of NMR. Advanced NMR instruments and applications tend to be clustered in large, well-established research universities, while less well-equipped institutions may struggle to find appropriate instrumentation and expertise. In short, advanced NMR is not as widely accessible as it could be and should be.

Lowering the barriers to discovery and application of NMR methods is needed to fully realize the potential impact and utility of NMR spectroscopy for addressing challenging problems in material science, metabolomics, structural biology, chemistry, and other applications. This need for democratization applies to both experts and nonexperts. The Network for Advanced NMR (NAN) is an effort to democratize discovery and access to NMR resources, simplify and standardize use, and liberate data for community access and reuse. Here we describe the design and implementation of key components of the NAN cyberinfrastructure: the NAN data transport system (NDTS), the NAN data browser, and the virtual NAN Operations Center (vNOC). Forthcoming publications will describe NAN knowledgebases, comprised of tested protocols and example data for experiments in solution and solid-state structural biology, metabolomics, and materials science.

## Barriers to Wider Application of NMR

Although NMR spectroscopy is incredibly versatile, procedures for preparing samples and for collecting, processing, and analyzing data are very complex. This complexity, along with a lack of standardization and infrastructure for data sharing, has kept NMR largely within the domain of NMR specialists. One of the goals of NAN is to standardize and document NMR methods to make the technique more accessible to non-specialists. NAN addresses many of these barriers:

### Instrument discovery

High-field NMR instruments, in particular, are generally found in shared facilities that operate as regional or national resources. NMR instruments can be configured in many ways, with instruments configured for one class of applications (e.g. solution, solid-state, micro-imaging, *in vivo*) typically limited to that single specific application. Finding a suitable instrument for a particular application can be difficult because there is no central clearinghouse with information about instrument configurations at all regional or national NMR facilities.

### Sample preparation

Sample conditions have a dramatic impact on the quality of NMR data that can be attained. Specialist laboratories have a wealth of experience, but the need for help with sample preparation is the second hurdle encountered by non-specialists (after determining what NMR technology is appropriate for the application).

### Instrument operation

Part of the versatility of NMR comes from the pulse sequences used to elicit responses from nuclear spin systems designed to reveal physical or chemical characteristics of the sample. Early pulsed NMR instruments were literally “hard-wired” to perform a limited set of pulse sequences, but modern spectrometers universally use software to determine the sequence of pulses, making it possible to instantly implement one of thousands of possible experiments. Unfortunately, the result has been a “library of Babel”^6^ of pulse sequences, some distributed by the instrument manufacturers, others developed and distributed by academic laboratories. As with NMR hardware, there is no central clearinghouse for pulse sequences. Exacerbating the complexity, many pulse sequences lack documentation or metadata needed to understand or use them effectively.

### Reproducibility

Scientific progress relies on the ability to reproduce results. In NMR, reproducibility demands detailed metadata encompassing sample provenance and conditions, instrument configuration, experiment design, and data processing parameters. Even when empirical NMR data are shared as supplementary material associated with publications, the metadata needed to fully replicate the experiment are often missing. Additionally, the lack of long-term data persistence further hampers reproducibility.

### Open science

Funders of scientific research worldwide are increasingly mandating public sharing of data that support published findings. Researchers must navigate a growing array of options for data sharing and preservation, including specialist repositories, generalist repositories, and journal websites. However, in the absence of standards for data provenance, formats, or metadata, shared data may be persistent but neither findable nor reusable. Researchers remain largely responsible for developing their own data management plans in response to these mandates.

## FAIR Principles and Data Sharing for NMR Data

Making NMR data “FAIR” (Findable, Accessible, Interoperable, and Reusable)^7^ is a central goal of NAN. The platform supports these principles as follows:

### Findable

NAN allows users to locate datasets by name or date, similar to conventional file browsers, and supports advanced search based on dozens of NMR-specific metadata tags. Published datasets are assigned persistent identifiers (PIDs) to ensure long-term discoverability.

### Accessible

NAN makes NMR data available via the web from any internet-connected device. Users can also transfer datasets directly to their NMRbox^8^ accounts for further processing and analysis.

### Interoperable

NAN harvests and standardizes metadata from nearly all existing versions of Bruker and Varian/Agilent instrument software, facilitating cross-platform use and integration.

### Reusable

In addition to experimental data, NAN collects pulse sequences, RF waveforms, and instrument parameters, providing the complete context needed to accurately reproduce the experiment.

Federal funding agencies require researchers to submit data management and sharing plans that describe how scientific data will be handled and shared. These agencies also mandate that data be made publicly available as soon as possible, and no later than the publication of associated research findings. Despite this, most NMR research has not made raw time-domain data accessible to the public, in part because building the necessary infrastructure is non-trivial. General-purpose scientific data repositories have emerged as a compliance option for researchers and institutions. However, these repositories often lack domain-specific modeling, resulting in a fragmented ecosystem where entries are difficult to query. This challenge is compounded by the fact that such repositories are distributed across different platforms and institutions, making federation nearly impossible without a shared metadata framework. NAN addresses these issues by providing a rich data model for time-domain data that captures detailed metadata about the user, sample, facility, instrument, and experimental setup, providing a wealth of metadata tags that are easily searched. The Biological Magnetic Resonance Data Bank (BMRB^9^) accepts raw time-domain data; however, the BMRB is limited to NMR data on biological molecules and systems.

## Methods

The NAN cyberinfrastructure is designed to streamline data acquisition, management, and access for NMR facilities, ensuring seamless integration of experimental data with metadata, storage, and user access controls. It consists of multiple interconnected components that facilitate automated data harvesting, secure storage, flexible and robust permission management, and real-time operations monitoring. This section provides an overview of the architecture and functionality of these components, highlighting their role in enabling efficient data organization, sharing, and analysis.

### Overview of the NAN Cyberinfrastructure

The NAN cyberinfrastructure consists of four main components:

1. The **NAN data transport system (NDTS)**
2. The **NAN archive**, which includes the NAN database and file storage
3. The **web portal**, featuring the **data browser** for viewing and managing samples, datasets and collections; **dashboards** for **facility** and **lab administration** management; and tools for managing ultra-high field instrument access to a few specific instruments
4. **Real-time monitoring** through a virtual NAN Operations Center

The cyberinfrastructure enables the automated harvesting of experimental data from NMR spectrometers, linking it with metadata on the facility, instrument, probe, experimental parameters, user identity, projects, and samples. The cyberinfrastructure provides robust data storage with disaster recovery and fosters data sharing and reuse. Users can add additional metadata, such as notes, tags, and sample metadata, both at the spectrometer and through the web portal, and can attach supplemental data, including processed data and unique identifiers for associated data from other repositories or associated publications. The system offers a fine-grained permission model, easy metadata visualization and download through data and sample browsers, and tools for organizing data. Additionally, it supports public access to data, publishing immutable and linkable persistent copies of data, and integration with the NMRbox platform. The system empowers NMR facility managers with powerful tools to manage data collected in their facilities and provides them with rich metrics and tools for report generation on operations through the vNOC.

### Accounts & permissions

To access non-public resources of NAN, users utilize a single sign-on (SSO) service hosted on NMRhub.org, which is shared between NAN and NMRbox. NMRhub account owners must provide their ORCID^10^ and indicate their principal investigator (PI) or declare themselves as a PI. All accounts and PI status are verified by NMRhub staff, while PI assignments initiated by users are validated by PIs or their delegates (see below). Non-public features of the NAN portal are disabled for users until a PI is designated and validated. When a new NMR facility joins NAN, an initial facility manager is added administratively by NAN staff. Subsequently, additional facility managers and staff are managed by existing facility personnel through the NAN web portal.

**Fig. 1:**
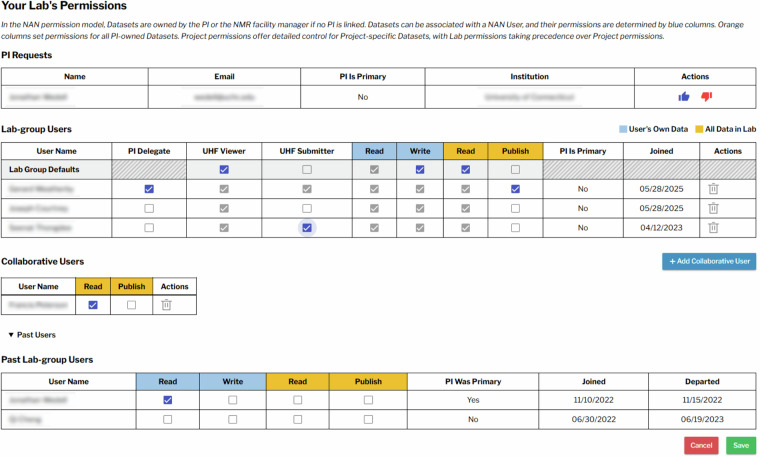
Permission matrix for data access in the NAN data browser. Screenshot of the lab-permission matrix, where PIs or their delegates can manage PI requests, control read/write privileges for current and former lab members and collaborators, assign UHF access, and designate additional delegates. Individual and lab-wide data access can be configured separately, with global defaults available for lab-wide settings.

Data harvested by NAN is associated with the NAN user who collected the data, but the PI is the responsible party, who acts as proxy for their institution. Users associated with a PI automatically appear on the **lab permissions** dashboard for PIs (see Fig. 1). PIs have comprehensive control over lab member permissions, including the ability to manage *read* and *write* privileges for data associated with a specific user. PIs can also regulate *read* and *publish* permissions for all lab data, as well as for external collaborators (individuals outside their lab group). While fine-grained permissions for individual users are supported, the PIs can set lab group default project permissions for all lab members to ease the administrative burden. PIs may remove users from the current lab group, but they remain linked to the PI as a past user. Permissions for former lab members can be managed in the same manner as active group members. Users can see all data for which they have viewing permissions, which always includes all public and published data. Permissions can also be associated with specific *projects* allowing additional permissions to be assigned to specific lab members, and collaborators can be granted access to project data without gaining access to all lab data.

PIs can assign *PI delegates* to handle administrative tasks. Delegates can switch between their roles as user (lab member) and PI delegate. In the PI delegate role, they can act as the PI to perform tasks such as accepting PI requests, managing lab members permissions, and controlling data access.

Facility managers serve as administrators for data collected within their facilities. They have the authority to view datasets generated at their site and to manage data access, particularly in cases where no NAN user or PI is associated with a dataset at the time of data acquisition and harvesting, or when the NAN user needs to be reassigned after harvesting. Through a web dashboard, facility managers can manage the link between spectrometer workstation users and NAN accounts, set the default data harvesting mode, and define whether users are allowed to change the harvesting setting or specify a different NAN username. Facility managers also can remove datasets that were harvested when they should not have been.

### Data security & trust

While no system can guarantee perfect security or trust, NAN employs a layered strategy combining technical safeguards with controlled administrative workflows. Access is restricted through role-based Active Directory groups, and key-based SSH is required for all logins. Code changes are version-controlled in Git and can be rolled back if needed. Systems are continuously monitored with the CrowdStrike Falcon agent, and server logs are centrally aggregated for auditing. As an NSF Trusted CI Center of Excellence, NAN undergoes periodic third-party reviews and aligns its policies with research cyberinfrastructure best practices.

All datasets are stored on a fault-tolerant Dell PowerEdge storage appliance with active monitoring. Daily snapshots enable recovery from accidental deletion. A disaster recovery copy is written to a WORM (Write Once Read Many) S3 bucket on a Scality RING cluster, with quarterly renewable retention leases preventing modification or deletion, even by administrators. Leases renew automatically unless a dataset is flagged to be purged. Object versioning maintains a complete history of dataset changes. Additionally, a separate system administrator maintains an independent copy on a physically and logically separate system, ensuring no single individual can destroy all data.

The PostgreSQL database replicates in real time to a secondary datacenter, with daily backups enabling full recovery. Critical services are distributed across two restricted-access datacenters to eliminate single points of failure. Changes to dataset metadata are captured in immutable audit tables, preserving a complete change history.

### Data harvesting (NDTS)

**Fig. 2:**
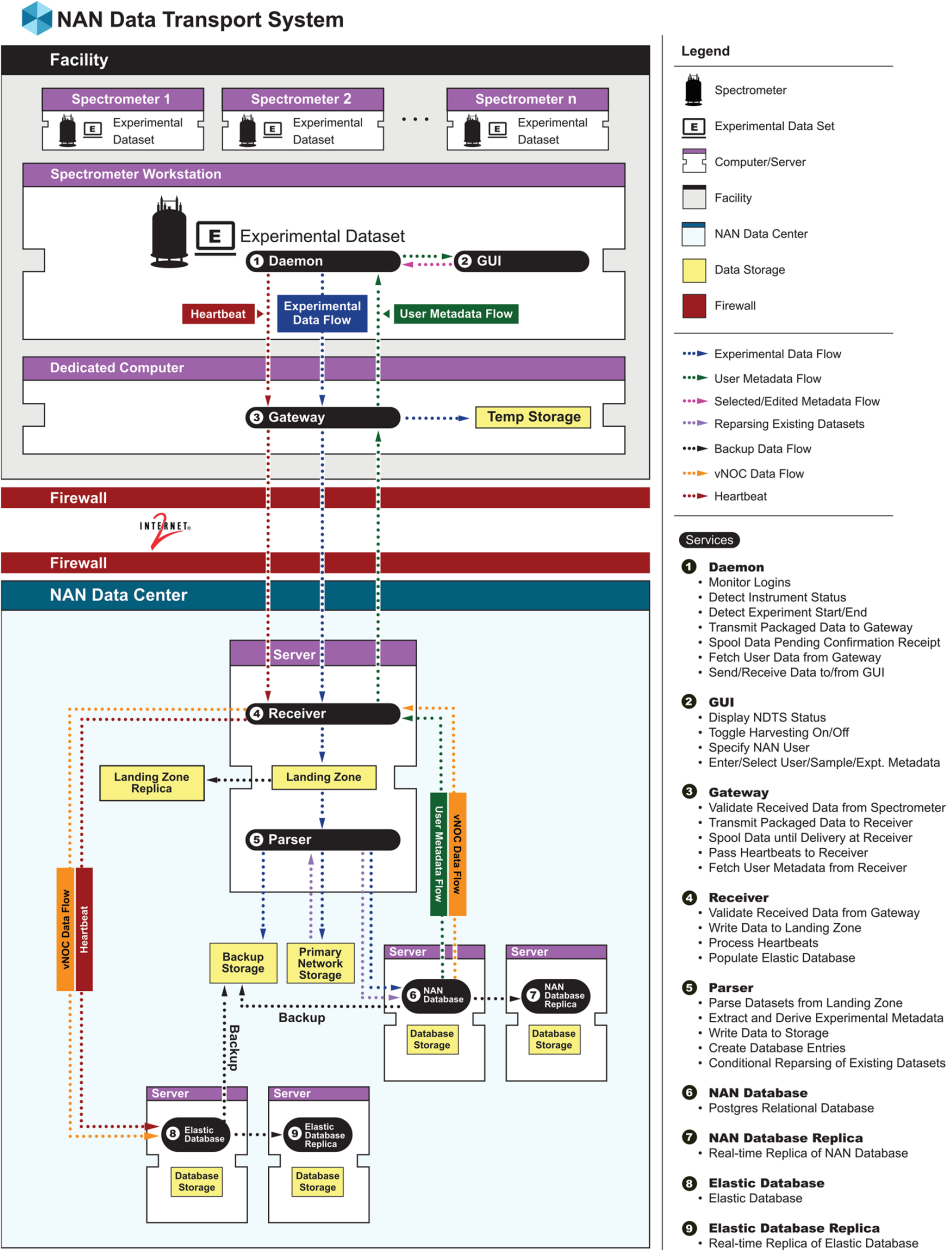
NAN Data Transport System (NDTS) Data Flow and Architecture. Schematic illustration the flow of experimental data, metadata, and system heartbeats within NDTS. The diagram depicts only a single NMR facility node. In addition to metadata associated with experimental datasets depicted by blue arrows, metadata from the NAN portal (e.g. NAN user list, local workstation to NAN user associations, users projects, studies, and samples, default NDTS settings for users) is conveyed to the spectrometer workstation.

**Fig. 3:**
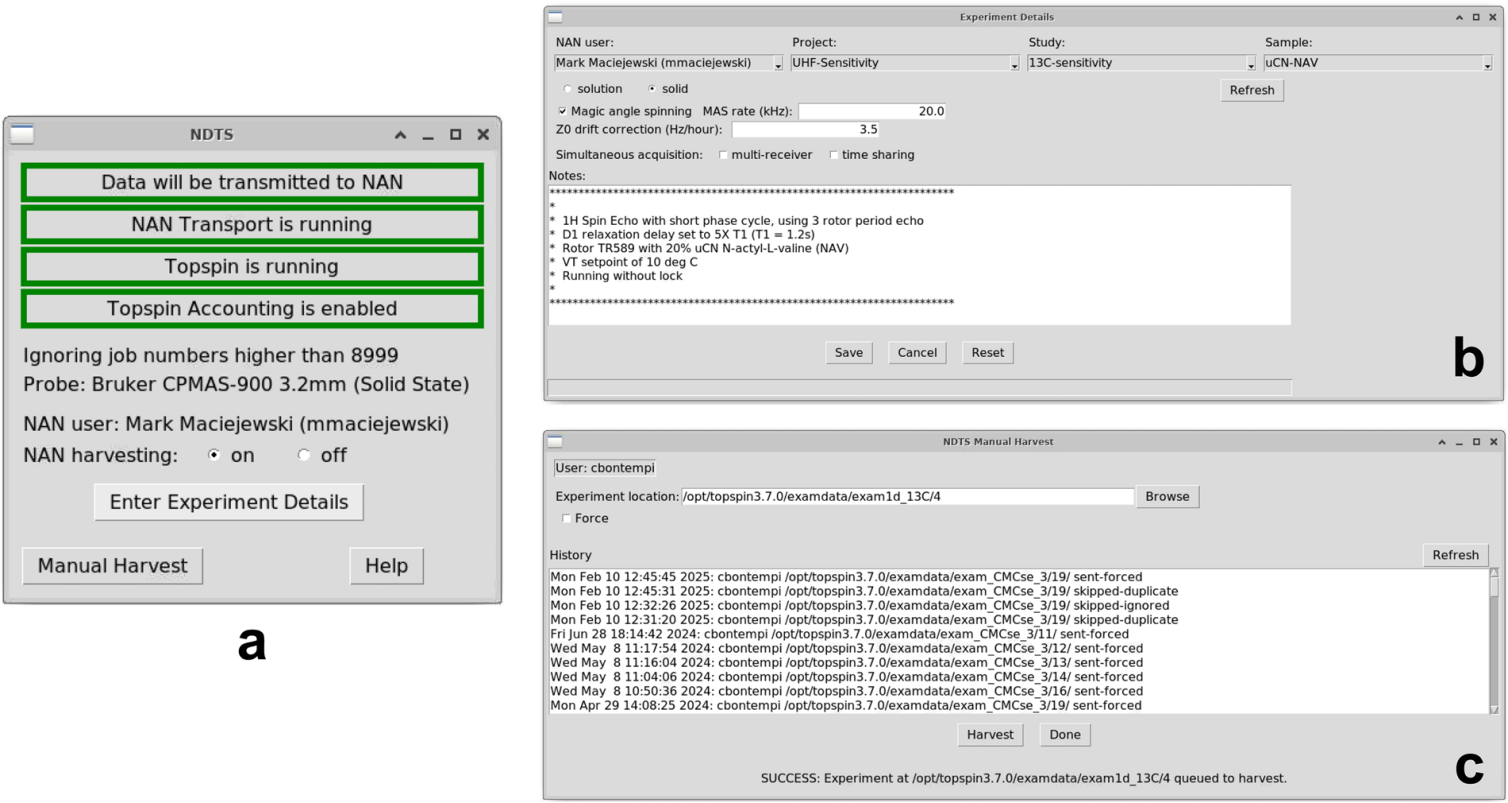
NDTS GUI and Experimental Details. (**a**) Screenshot of the NDTS GUI showing the system status, selected NAN user, and installed probe. Radio buttons allow authorized users to enable or suspend NAN harvesting, based on facility permissions. (**b**) Experimental details panel, where users can switch the active NAN user, select a project, study, and sample for dataset association, and enter additional metadata that is difficult to capture accurately in parameter files. (**c**) Dialog panel for manual data upload, for harvesting data collected prior to the instrument being connected to NAN, or for data not harvested because NDTS status was off.

NDTS consists of four main components: the daemon, graphical user interface (GUI), receiver, and parser. Figure 2 illustrates the data movement in NDTS; technical details of the NDTS components are provided below. The daemon detects when an experiment completes and automatically harvests all files necessary to recapitulate the experiment. It is aware of the relevant file locations for different versions of vendor-specific software (VnmrJ and TopSpin). The daemon harvests time domain data, parameter files, waveform files, non-uniform sampling (NUS) schedules, pulse program files, probe head files, and includes the entire content of the experiment directory. In addition, the daemon harvests metadata including the local workstation *username*, *facility*, *instrument*, and installed *probe*. The NDTS GUI, shown in Fig. 3a, displays NDTS status, the *NAN user*, and the *installed probe* and allows the user to change the harvesting condition (on or off, if they have appropriate permission). If the harvesting condition is *off*, no data is transferred, allowing the facility to strictly control data handling and access for any proprietary projects. Through the **experiment detail** page (Fig. 3b), users can update the *NAN user* and manually add or link existing metadata such as *project*, *study*, *sample*, *notes*, and experimental parameters that are not accurately captured in the instrument parameter files. The NDTS GUI provides a **manual harvest** button, shown in Fig. 3c, to harvest any experiment after the acquisition has been completed allowing data collection in cases where harvesting was disabled by accident or to harvest datasets that existed prior to NDTS being installed. All harvested data is sent by the daemon from the spectrometer workstation to the facility gateway. Local caching on the spectrometer workstation ensures that no data is lost even if the connection to the gateway is temporarily lost. By default, the cache is cleared once data arrival at the receiver is confirmed. An optional capability of the gateway is to serve as a local archive for all data emanating from the facility. This feature not only provides additional data security but can also address data patriation regulations that apply in some geographies. The daemon keeps detailed logs of all actions for auditing and debugging.

All datasets are associated with a *session ID*, with new sessions being created when a user logs in, the NMR spectrometer software (VnmrJ or Topspin) is started, or the selected NAN user in the NDTS GUI is changed. Datasets are named based on the directory where they are collected (e.g. exp*N* for VnmrJ, and data_directory/*N* for TopSpin). It is possible, and even likely, that the same experiment is run multiple times during a session, as initial calibration experiments or 2D projections are often conducted before the full experiment. Metabolomics or ligand-binding experiments can have hundreds or thousands of the same experiment applied to numerous samples. Use cases in biological NMR, especially in the solid-state, often require extensive calibration and optimization datasets. NDTS automatically harvests all completed experiments, which may result in the collection of “redundant” dataset names within a session. By default, the last experiment in a set of redundant dataset names is tagged as “preferred”, while all others with the same name in that session are tagged as “redundant”. Users can change the redundant/preferred status, and an icon on the preferred dataset indicates if redundant datasets exist, the number of redundant datasets, and can be clicked to open a detailed view of the redundant datasets. In this way the primary data browser view provides an uncluttered view, free of redundant datasets, while allowing easy inspection of the set of redundant datasets when needed.

The facility gateway is a dedicated computer, configured with a modern operating system with security patches, co-located in the same network as the spectrometer workstations. It acts as a bridge between the spectrometer workstations and the NAN archive, serving as the only computer that connects outside the local facility. The gateway securely transmits harvested datasets to the NAN receiver as well as periodic heartbeats for status monitoring and will locally spool data if the receiver cannot be reached (for example, in case of internet outage). The gateway also requests updated facility settings and user data (*facility users*, p*rojects*, *studies*, and s*amples*) from the receiver every 10 minutes, which is then pulled by the daemon on the spectrometer workstations ensuring that all settings and metadata remain current. Users can force a refresh from the NDTS GUI to pull information, such as samples, immediately to the spectrometer workstation rather than waiting for the 10-minute automatic pull.

The receiver, located in the NAN datacenter, is a network service that accepts transmitted experiment data from facility gateways, ensuring guaranteed reception of each experiment. It also handles packaging of facility manager settings and user data from the NAN portal that will be fetched by the gateway computers and responds to spectrometer heartbeat messages. Data collected by the receiver is sent to the parser which scans each dataset, derives additional metadata based on experimental parameters, creates database entries with corresponding metadata, and saves the dataset files to the NAN primary and disaster recovery storage. The parser can re-scan any experiment to update or correct metadata without disrupting the repository or to back-fill metadata in existing datasets if additional features are added to the parser. When re-parsing, no prior data is over-written.

The receiver/parser is also responsible for populating the Elastic^11^ database used to drive the vNOC. Heartbeats from the NMR spectrometers are processed by the receiver to extract instrument status information, which is then entered in the Elastic database^11^. Beyond status information provided by the heartbeats, the vNOC also provides detailed usage metrics (see below) with data being queried from the NAN PostgreSQL database and pushed to the Elastic database at timed intervals.

### Experiment metadata

While capturing experimental dataset files is critical, the ability to search, organize, and utilize the data is dependent on the metadata associated with the datasets. NDTS automatically collects an extensive list of metadata including *dataset name, unique ID, workstation user*, experiment *date* and *time, facility, instrument, field strength, state* (solution/solid), *pulse program, redundancy status, title, and transfer mode*. From the NDTS GUI users can define metadata that is difficult, or impossible, to capture automatically such as *temperature*, *project, study, sample, magic-angle spinning (MAS) rates, magnetic field (Z*_*0*_*) drift rate*, and *notes*. To ensure consistent search and aggregation, NAN normalizes parameter values by using controlled lists, use alias mappings for a subset of experimental parameters from different vendors, enforcing fixed unit for numeric entries, and applying type checking in the front and back end. Select elements from parameter files are parsed into normalized and searchable terms and the parameter files are saved as JSON objects in the database. In addition, heuristics are utilized to derive additional experiment metadata such as *dimensionality, nuclei*, and *direct nucleus* (directly detected nucleus). Users, or facility managers, can update certain data that may be incorrect or incomplete, while preserving original values that can be reverted if necessary.

### Sample metadata

Most of the experimental metadata described above is determined automatically with minimal user input. However, details about the sample in the spectrometer must be provided by the user. The NAN portal is designed to be agnostic to the type of NMR, enabling users to input sample metadata across a wide range of NMR applications. This sample information can be entered in the NAN **sample browser**, which is within the data browser on the NAN portal. Importantly, samples can be created in the NAN portal at the time that they are created at the lab bench and before any NMR data has been acquired to maximize accuracy and completeness in the sample description. If the sample is created before data collection, users can select the correct sample in the NDTS GUI. Users can also associate samples with datasets in the NAN data browser at any time after data acquisition. Numeric sample attributes such as pH, temperature, and concentration are entered in defined units and stored as typed numeric values, ensuring consistent normalization across samples.

#### Sample definition

To address the complexity of entering sample information, the NAN portal provides a form-based sample definition tool that is divided into five tabs: **sample**, **container**, **composition**, **sample properties**, and **additional information**, and there are note sections for: **general**, **sample preparation**, **container**, and **composition**. While the sample definition form is comprehensive, the only required fields are the *PI, sample name*, and sample *state* (solution or solid) which is a necessary distinction as the forms differ between the two states. Users can edit sample information after sample creation in the browser, allowing data to be entered across multiple sessions. The system uses groupings and controlled vocabularies where possible to promote consistency and searchability, while still allowing free-form entries when needed to accommodate the diverse range of sample definitions that users will require.

The **sample** tab allows users to input the *PI, sample name, state, preparation date, description*, and *tags*. Users may also provide a URL linking to external sources, such as Protocols.io or a laboratory information management system (LIMS) to link the sample preparation protocol. The **container** tab captures information specific to the sample container, with different fields for tubes and rotors. For tubes, users specify the tube *label, geometry*, and *outer diameter*. For rotors, users input details such as *rotor type, label, material, cap material, size*, and information on *spacers, packing*, and *sealing method*. The **sample properties** tab varies depending on the sample state. For liquids (solution state), users can record *pH* (including measurement method), *volume*, *ionic strength*, *conductivity*, *alignment*, and *paramagnetic properties*. For solid-state samples, users can provide the *mass*, *ionic strength*, *hydration level*, *conductivity*, and *paramagnetic properties*.

**Fig. 4:**
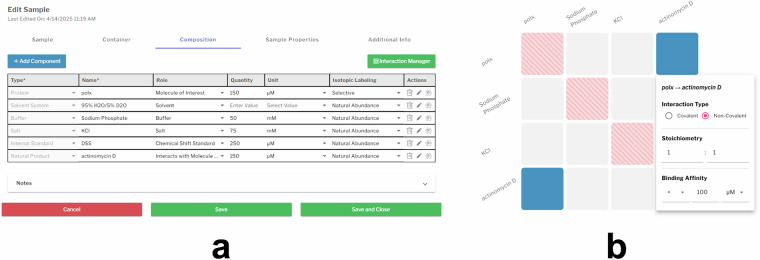
Sample definition tool and sample component interaction matrix. (**a**) Screenshot of the sample definition tool showing the Composition tab, where components can be added or removed and defined by type, name, role, quantity/unit, and labeling. Pencil and atom icons open detailed panels for specifying component type and isotopic labeling. Additional sample information can be entered on the Sample, Container, and Sample Properties tabs. (**b**) Interaction manager panel for defining covalent or non-covalent interactions between components, including stoichiometry and binding affinity.

The **composition** tab is where users specify the components present in the NMR sample. Each component is displayed in an editable table, allowing users to define the *type, name, role, quantity, unit*, and *isotopic labeling* (see Fig. 4a). Component types are organized into high-level categories, with each type linked to a details page containing fields relevant to that component (see Table 1). Table 2 lists the additional fields for each of the eight details pages listed in Table 1. The component *name* field permits easy identification and is free-form text except for the following component types; *solvent system*, *buffer*, *salt*, *internal standard*, *detergent*, *lipid*, and *paramagnetic compound*, which have controlled vocabularies. The *role* field is a multi-select option for defining the component’s function in the sample and includes *molecule of interest, solvent, buffer, salt, chemical shift standard, detergent, excipient, polarizing agent, paramagnetic agent, alignment medium, membrane mimetic, interacts with molecule of interest*, or *other*. Users can define the *quantity* and corresponding *units* in terms of *concentration, mass*, or *volume*. An interaction manager tool allows users to define interactions between components in the sample (see Fig. 4b). The interaction matrix lists all components along both axes, allowing users to specify interactions by selecting a cell. Selections are mirrored automatically across the diagonal. Users can define *interaction type* (covalent or non-covalent), *stoichiometry*, and *binding affinity*.

**Table 1:** High-level component categories, component types, and associated details page.

Category	Type	Details Page
Bio Macromolecule	Protein	Proteins
	Nucleic Acid (DNA)	Nucleic Acid
	Nucleic Acid (RNA)	Nucleic Acid
	Carbohydrate	Carbohydrate
Small Molecule	Metabolite	Chemical Formula
	Natural Product	Chemical Formula
	Other Organic	Chemical Formula
Chemical Formula	Chemical Formula	Chemical Formula
Polymer	Polymer	Polymer
Mixtures	Biological Material	Biological Material
	Environmental Sample	Environmental
Additives	Detergent	Component
	Lipid	Component
	Micelle	Component
	Bicelle	Component
	Nano-disk	Component
	Stretched or Compressed Gel	Component
	Excipient	Component
	Polarizing Agent	Component
	Paramagnetic Compound	Component
Components	Solvent System	Component
	Buffer	Component
	Salt	Component
	Internal Standard	Component
	Referencing Standard	Component
Other	Other	Component

**Table 2:** Field definitions and controlled vocabulary used across the sample component details pages.

Details Page	Fields and controlled lists
protein	name, role, quantity/unit, molecular weight (MW), purity, UniProt ID, # subunits, conformational state (globular, membrane, fibril, disordered), monomer/homo-oligomer (symmetric/asymmetric), AA sequence, modifications (linkage, post-translational modifications [phosphorylation, acetylation, glycosylation, lipidation, nitrosylation, ubiquitination], non-standard AA, paramagnetic tags, purification tags [N-His6, C-His6, N-Strep, C-Strep, N-GST, C-GST, N-thioredoxin, C-thioredoxin])
nucleic acid (DNA/RNA)	name, role, quantity/unit, MW, purity, GenBank ID, sequence, non-standard description, covalent linkage description, modifications (linkage, post-translational modifications [phosphorylation, acetylation, glycosylation, lipidation, nitrosylation, ubiquitination], non-standard AA, paramagnetic tags, purification tags [N-His6, C-His6, N-Strep, C-Strep, N-GST, C-GST, N-thioredoxin, C-thioredoxin])
carbohydrate	name, role, quantity/unit, MW, purity, classification (oligosaccharide/polysaccharide), IUPAC-condensed string, tags, modifications (glycopeptide, glycolipid, glycosides)
polymer	name, role, quantity/unit, purity, tacticity (atactic, eutectic, isotactic, syntactic, indeterminate), branching (linear, branched), source (synthetic, biological [biological source]), weight average MW, # average MW, dispersity, # of subunits, degree of substitution, length of repeated sequence, total # of repeats, sequence order, define polymer subunit(s)
biological material	name, quantity/unit, source organism (human, mouse, *E. coli*), biological type (serum, urine, whole blood, cell extract, tissue), sample category (study sample, buffer blank, extraction blank, internal QC, external QC), local sample ID, extraction method (none, methanol:water, methanol, water, isopropanol, DMSO, chloroform:methanol, other), description
environmental sample	name, quantity/unit, source location, source class/material, geo location, sample category, local sample ID, extraction type, description
chemical formula	name, role, quantity/unit, MW, purity, CHEBI ID, CAS ID, PubChem ID, chemical formula, InChi String, class (small molecule, polycrystalline, amorphous, zeolite, single crystal, heterostructure, glass)
component	name, role, quantity/unit, MW purity

The *isotopic labeling* field provides a dropdown menu with common labeling types, such as *natural abundance, U-13C, U-15N-13C*, and more. An advanced isotope editor allows for further customization, including site-specific labeling, amino acid-specific labeling, and labeling based on metabolic precursors.

#### Cloning, bulk sample definition, and dataset mapping

Defining sample metadata can be tedious and time-consuming. To simplify this process, NAN offers three key features: an intuitive interface, a sample cloning option, and a multi-sample creation tool. Often, users need to create multiple samples with only minor differences, as for data collected on a mutant versus wild-type protein or apo- versus ligand-bound protein. To expedite this, the context menu (launched from a right mouse click) in the sample browser provides a *clone* option, prompting the user for a new sample *name* and placing the browser in edit mode to modify the cloned sample information. For scenarios requiring many samples, such as titration series or metabolomics studies, users can utilize the multi-sample creation tool. In this mode, users utilize the same intuitive interface and complete the sample information for invariant fields, as they would for a single sample, and identify fields that vary across the samples. The user then provides a *collection name*, *base sample name*, and *number of samples* and the system generates a table with rows for each sample and columns for the variable fields. Users can edit the table directly, upload a pre-filled column separated value template, or reorder, remove, and add rows. Once finalized, all sample records are created and organized within a named sample collection. Sample names are automatically created using the base sample name provided and appended with an underscore and a three-digit sample number based on the order of the sample in the table.

Users can quickly assign samples to datasets using the data browser context menu. However, mapping individual samples to datasets from a large collection created with the multi-sample creation tool can be labor-intensive. To streamline this process, a mapping tool is provided for efficient sample-to-dataset associations. The tool displays two columns: samples on the left and datasets on the right, with arrows allowing users to link samples to their corresponding datasets. Once mappings are configured, users can finalize the process with a single action. The tool supports flexible mapping scenarios, including one-to-one relationships when the number of samples matches the datasets and one-to-many relationships when multiple datasets are collected per sample. It accommodates cases where several datasets are generated per sample inserted into the magnet or when samples are processed through a single experiment in successive cycles. Ideally, the number of datasets would be an integer multiple of the samples, but this isn’t always feasible due to instrumentation or experimental issues. To address discrepancies, the mapping tool offers advanced features such as sorting samples and datasets by various criteria and inserting or deleting entries with automatic list adjustments when changes are made mid-list.

## Data and sample browsers

**Fig. 5:**
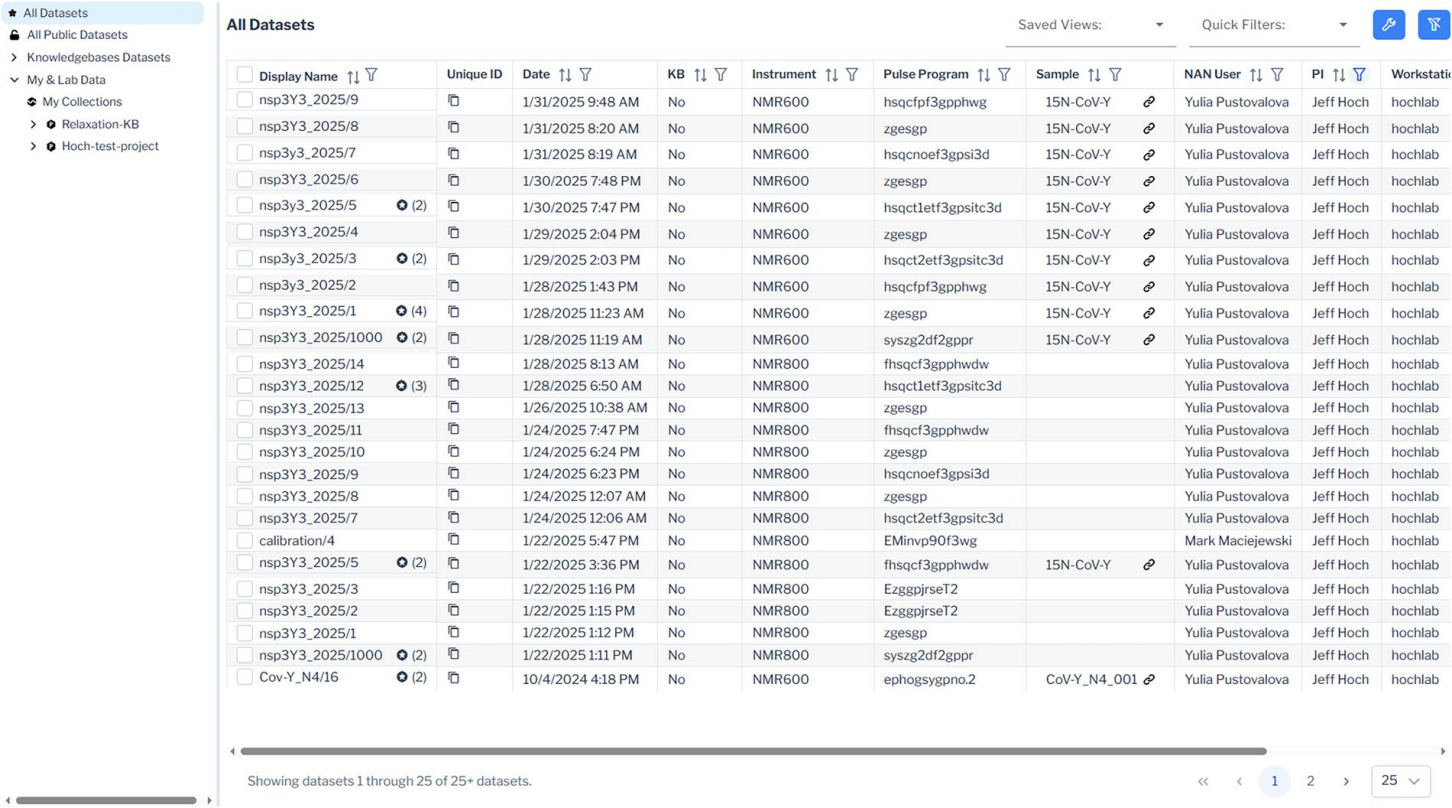
Data browser. Screenshot of the dataset browser, featuring a left-hand navigation pane for switching between All Datasets, Public Datasets, Knowledgebase Datasets, and My & Lab Data, as well as viewing projects, studies, and collections hierarchically. The main table displays datasets with sortable and filterable metadata columns; advanced searches can be performed by combining filters. Customization tools include Saved Views, Quick Filters, a clear filter icon (for both visible and hidden columns), and a wrench icon to select displayed metadata fields. Columns may be reordered, and all settings are saved as user preferences.

**Fig. 6:**
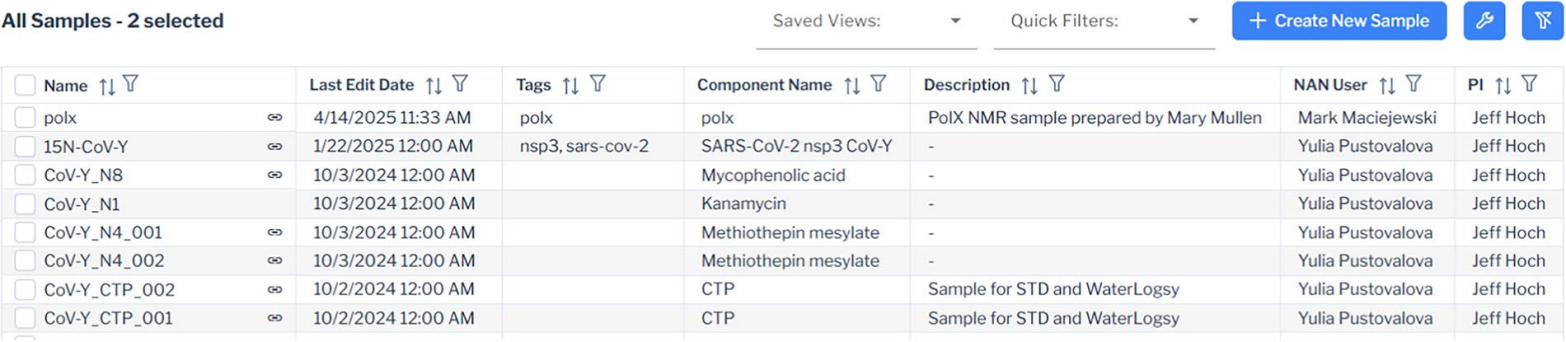
Sample browser. Screenshot of the sample browser, which displays samples in a sortable, filterable table. A link icon in the name column indicates samples linked to datasets and provides navigation. Users can customize visible metadata fields with the wrench icon, rearrange columns by dragging, and save their layout as a user preference. Samples are sorted by last edit date by default, with options for column-based sorting and advanced filtering. Saved Views, Quick Filters, and a clear filter icon support efficient filtering and search replication.

The data browser allows listing of all datasets and samples (own, lab, and collaborator) that the user has permission to view, as well as all public and published data. A **my permissions** menu item provides the ability for a user to view their permissions for lab group and project data. Datasets and samples are displayed in a table format with 25, 50, 100, or 500 rows with pagination if the number of rows exceeds the value being displayed (see Figs. 5 and 6 for the data and sample browsers, respectively).

For datasets, up to 37 columns may be displayed corresponding to many of the metadata fields associated with NMR datasets such as a *date* collected, *facility, instrument*, *pulse program*, *sample*, *PI*, *NAN user*, *workstation user, tags, notes*, and a host of experimental parameters. The *display name* is fixed as the first column, but all other columns may be toggled on and off and re-ordered, allowing the user to customize their preferred view. The current view is saved as a user-specific default so that the user will return to the same view the next time they log in. The *display name* is set automatically based on the *dataset name* but can be edited by the user to be shorter or more meaningful, as needed. Datasets which are part of a redundant collection are identified with an icon badge that includes the number of redundant datasets in the redundant set. An expanded redundant view allows all datasets to be viewed for a given redundant dataset collection. Link badges for samples and datasets allow easy navigation between datasets and their linked sample or from the sample to experimental datasets with the linked dataset.

The sample browser follows the same format used for dataset browsing with samples viewed in a table with up to twenty columns including *name*, *last edit date, NAN user, PI, description, tags, status, container type, components*, and *pH*. Each column may be toggled on/off or reordered except the *name* field which is fixed as the first column.

### Filtering and searching datasets and samples

Each column in the dataset and sample browser may be sorted and filtered by one or more filters. Combinations of filters can produce advanced searches and can be saved with a user defined name for easy future use. A collection of quick filters is provided to expedite filtering for common items such as *my data* or *hide inactive samples*. The filter icon in the top right indicates whether filters are applied to visible or hidden columns and provides an easy way to clear them.

### Editing dataset and sample metadata

Dataset metadata can be edited by double-clicking a row in the data browser or selecting *edit dataset* from the context menu, which opens an experiment detail modal window. Fixed fields, such as experiment date, instrument, and workstation user are non-editable, while fields like redundant status, dimensions, and nuclei (interpreted by the parser) remain editable for corrections. Similarly, samples can be edited via double-click or the context menu, with all fields being modifiable. Editing permissions depend on user roles: read-only users can view but not modify fields, while users with edit privileges can update editable fields. For efficiency, bulk editing is available for groups of datasets or samples sharing common attributes, reducing the burden of individual modifications, especially handy if the number of datasets or samples is large. Metadata changes are tracked in a provenance record as described below.

### Dataset and sample context menu

The data browser provides an extensive set of possible actions via a context menu (accessible by a right mouse click), enabling users and facility managers to manage, access, and annotate datasets efficiently. Actions unavailable due to permissions or applicability appear grayed out. The browser supports multi-selection using standard keyboard shortcuts allowing bulk actions where appropriate, see Table 3.

**Table 3:** List of the actions and their descriptions available from the dataset and sample browsers context menu.

Action	Description
***Dataset Browser***	
View / Edit Dataset	Opens a modal window to view or edit a dataset.
Reassign^a^	Assigns a dataset to a *NAN user* or changes the *NAN user*. Facility managers can reassign datasets to arbitrary users with no time constraints. Standard users can reassign datasets within their lab group for up to three months after harvesting.
Download^a^	Enables datasets to be downloaded (for processing, analysis or local archive) in different organizational layouts.
NMRbox Integration^a^	Copies a dataset from the NAN archive to a user’s NMRbox home folder in a predefined location (for processing and analysis) and allows retrieval of files (such as processed scripts or processed spectra) from the dataset’s post-acquisition directory on NMRbox back into the NAN archive.
Supplemental Data	Add or view supplemental data associated with a dataset.
Redundancy^a^	Modifies the *redundancy status* of a dataset as “preferred” or “redundant”. By default, the last experiment of a redundant set is marked as preferred and others as redundant.
Link Sample^a^	Links a sample to a dataset.
Classification^a^	Classifies a dataset as a *calibration experiment, failed experiment (*due to sample, instrument, or setup issues), *test experiment*, or *successful experiment*. Facility managers can also mark datasets to be purged from the NAN archive if harvested in error.
Deposit to BMRB	Initiates a new BMRB deposition prepopulating information about the user, datasets, instruments, and sample information.
Tags^a^	Enables users to assign arbitrary tags to datasets.
Notes^a^	Allows users to add notes to datasets.
Unlink from Collection^a^	Removes a dataset from a collection.
Make Public^a^	Marks a dataset as publicly available.
Publish^a^	Publishes a dataset. This creates a permanent, un-editable copy with a unique identifier.
Copy Dataset Link	Copies the ARK URL to the clipboard for published datasets
***Sample Browser***	
View / Edit Sample	Opens a modal window to view or edit the sample.
Set Active Status	Sets the active status to active or inactive. Only active samples are shown when linking datasets or from the NDTS GUI.
Clone	Clones a sample prompting for a new sample *name* and opening the Edit Sample modal.
Delete	Deletes a sample. Note, samples linked to datasets cannot be deleted.
Unlink from Collection	Removes a sample from a collection.
Download XML	Downloads an XML record of the sample metadata.

^a^Actions that can be performed as a bulk action to multiple selected datasets where appropriate.

## Supplemental Data

NDTS harvests all files within an experimental dataset, along with additional files necessary to reconstitute the experiment such as waveform files, pre-compiled pulse programs, and others. However, there are relevant files and metadata information that are not present at the time of collection that may be useful to link with the dataset such as protocols, data processing and analysis scripts, links to external data sources such as BMRB entries, and others. To this end, NAN provides the ability to append this supplemental data to datasets and collections.

**Fig. 7:**
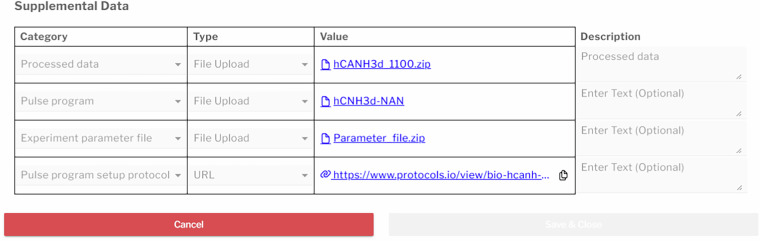
Supplemental data definition tool. Screenshot of the supplemental data modal, where users can add or view supplemental data by selecting a category and type (file upload, file in dataset, ID, or URL), entering a value, and optionally providing a description. Uploaded files become part of the dataset, while non-file entries are included in a CSV file upon download.

Supplemental data is added via a table in a **supplemental data** modal launched from the dataset editor or context menu (see Fig. 7). Each entry requires a *category*, followed by a *type*, *value*, and an optional *description*. Available types include *URLs, file uploads, files within the dataset*, and *ID numbers*. Categories include protocols for instrument setup, sample preparation, pulse program setup, signal processing, and analysis; scripts for experimental setup, signal processing, and analysis; ID numbers for BMRB, PDB, Metabolomics Workbench^12^ and generic DOIs; as well as data files for sample schedules, experimental parameters, pulse programs, processed data, and sample parameters. Supplemental data information becomes part of the dataset record and files are added to the dataset in a supplemental data directory. A list of supplemental data entries and files are included in dataset downloads. In the data browser, a *Supplemental Data* column identifies datasets that include supplemental material allowing filtering and sorting.

NAN and NMRbox share a common data infrastructure, allowing direct dataset transfers. From the context menu (right click) in the data browser, users can copy datasets to their NMRbox home folder, with copies tracked in the NAN database. If users generate new files in the **post-acquisition** directory on NMRbox, such as processed or analyzed data or processing scripts, they can use the *retrieve post-acquisition data* action to append these files to the original dataset in the NAN archive. Thus, the NAN data browser serves as a hub for linking raw NMR data and sample information to protocols, derived data (assignments, structure) and publications to facilitate retrieval of all the relevant information for a particular experiment.

## Projects, Funding, Studies, and Collections

NAN enables PIs to create projects, associate funding sources with them, and efficiently manage lab members and collaborators, including the option to grant all lab members automatic access to a project. Associating funding to a project is for attribution only; no billing occurs through the NAN portal. All users in a project can create studies within it and collections within studies. Datasets can be placed directly in projects, studies, and collections, providing a hierarchical data structure like a file system, where these entities act as folders. Ideally, most experimental datasets would be linked to a project, allowing funding sources to be directly associated with datasets and enabling powerful reporting for PIs and facilities. Users can select projects and studies from the NDTS GUI at the time of collection or drag and drop datasets into projects, studies, and collections from the data browser.

Projects and studies are created and managed at the lab group level, along with any collections within studies. However, collections can also be used independently of projects and are accessible in *my collections*, allowing users to organize their own data. Users’ collections remain private, though datasets within them adhere to the PI’s permissions.

## Dataset Categorization and Intelligent Filtering for Data Discovery

Datasets in NAN are categorized using a combination of automatic and user-defined methods to enhance data organization, retrieval, and analysis. These metadata fields include tags, pulse program, method of data harvesting, user-defined classifications, redundancy status, dimensionality, experimental parameters, linked samples, and supplemental data. Such structured categorization, along with additional metadata fields, facilitates the creation of powerful filters, allowing users to generate targeted sets of experimental data for download. As the volume of public datasets in NAN grows, these filters will become essential for researchers seeking specific subsets of data for automated data processing, machine learning training, and comparative analyses.

## Publishing & Public data

In NAN, “public” means that a dataset is accessible via the data browser to anyone (including users without NAN credentials), while remaining editable by authorized users. Although users can mark datasets as public at any time, NAN policy is to automatically mark them public three years after harvesting, with an option for users to extend the public release date by one year within six months of the release date. The public release date is clearly viewable and sortable. “Publishing” a dataset makes it public and creates a complete, immutable (i.e., uneditable) copy of the dataset (including its database entry, files, samples, and supplemental data) and assigns it a unique ARK^13^ persistent identifier. The ARK for a published dataset can be copied directly from the dataset context menu, and an ARK column in the data browser allows users to view and search by ARK. The original (parent) dataset remains fully editable, and a link is maintained between the published dataset and its parent. Each published copy is immutable, and if there is more than one published copy they are versioned (v1, v2, v3, etc.). If published again, the process is repeated, and all published versions receive a new version number. All published versions remain available to users: by default, the data browser shows the original (editable) dataset and the most recent published version, with options to view earlier published versions. Note that a dataset with no changes since the last publication will not be published again, even if requested by the user. Changes to datasets are stored in a provenance record. Dataset collections may also be published. When a collection is published, each individual dataset is published as described above, and the collection itself is assigned an ARK that resolves to the published collection in the data browser. This supports organization and simple citation of datasets when a paper is published or to meet funder requirements.

## Provenance Tracking of Metadata

A goal for the NAN archive is to utilize FAIR principles, with provenance tracking as a critical feature. Provenance tracking ensures that historical metadata is captured, enabling efficient searching and retrieval of both current and past metadata records. NAN maintains a record of all relevant metadata changes for datasets and samples where data interpretation or reuse would be affected, ensuring continued access without risk of loss or modification of the original files. Provenance data are efficiently captured using audit tables and triggers in PostgreSQL^14^, allowing logging of all metadata modifications and preserving a full historical record. Machine-readable metadata in XML format and a provenance record in W3C PROV^15^ format are generated on-demand at the time of download and embedded directly within each dataset, providing users with a self-contained package that includes all necessary metadata and historical context.

## Uploading Arbitrary Datasets

NDTS provides automated data harvesting from NAN-connected instruments, and the NDTS GUI includes a manual upload feature for cases where automatic harvesting was unintentionally disabled, or for data collected prior to connecting a spectrometer to NAN. When NDTS is installed on instruments, users may have datasets already stored on spectrometer workstations or lab computers that they wish to harvest to leverage NAN data organization and publishing features. While the NDTS manual harvesting feature allows users to upload individual experiments, it is not designed for bulk data uploads. To address this limitation, an arbitrary data upload tool with a structured workflow was developed:

1. Users create a tar/zip archive of the directory containing the data to be uploaded. The archive can have an arbitrary number of nested subdirectories.
2. The tar/zip file is uploaded to the NAN portal via a user NAN account.
3. A service on the NAN portal scans the file, identifying valid experimental datasets.
4. The identified datasets are presented in a staging table with columns similar to those in the data browser, allowing users to fill in required fields and edit metadata.
5. Once a dataset is ready, users select the datasets they wish to upload to NAN.
6. The selected datasets are sent to the parser and uploaded into the NAN archive.

The staging area is cached, enabling users to interact with the tool across multiple sessions and a full history of all arbitrary data uploads is kept. The system detects previously uploaded datasets using unique identifiers and notifies users of duplicates to prevent redundant entries. Datasets that do not meet minimum standards, such as those missing critical files or metadata, are rejected and only datasets from instruments registered in the NAN portal are accepted. Datasets uploaded via the arbitrary data upload tool will be recorded with a *transfer mode* of “arbitrary”.

## Virtual Network Operations Center (vNOC)

The vNOC is a set of dashboards that display the activity and operational status of NAN components. Each dashboard consists of multiple panels tailored for specific audiences. The data presented in vNOC falls into two main categories: facility status and statistics derived from heartbeat messages sent by the daemon on each spectrometer, and experiment statistics summarized from experiment records in the NAN PostgreSQL database. The dashboards serve two primary purposes, summarizing activity and identifying anomalies or unexpected behavior.

The vNOC dashboards provide insights at different levels of access. The **public** dashboard presents general NAN usage data without exposing sensitive information. It includes panels such as the names and locations of affiliated facilities and spectrometers, number of experiments harvested, number of users, and pulse programs. The **user** dashboard provides experiment statistics specific to the logged-in NAN user, displaying counts by facility, spectrometer, sample, project, funding source, and PI, as well as distinguishing between harvesting modes. The **PI** dashboard includes similar panels as the user dashboard but incorporating data from all users that have data assigned to the PI. The **facility manager** dashboard expands further by integrating facility-wide statistics, including spectrometer operational status. In addition to experiment data, it features panels that track spectrometer uptime, experiment rates per spectrometer, workstation usage, harvesting settings, and acquisition states. Facility managers can use this dashboard to detect issues with daemons or gateways that might affect data harvesting. The most comprehensive view is provided by the **administrator** dashboard, which consolidates data across all NAN facilities. In addition to the insights available in the facility manager dashboard, it includes operational metrics such as storage consumption. This dashboard is intended for system administrators who need to see system-wide performance and resource utilization.

A key feature of vNOC is its ability to filter data dynamically. Users can click on any facility, spectrometer, or other parameter within a panel to apply a filter across the entire dashboard. Active filters appear at the top of the interface and can be removed with a single click. Multiple filters can be applied simultaneously, allowing flexible data exploration. When users return to the dashboard, all filters reset to the default state. A specialized filtering option is the *time frame filter*, located in the upper-right corner of each dashboard. This filter provides a range of options, including quick selections (such as “Recently used”), absolute date ranges (e.g., “May 1st to May 22nd”), and relative time frames (e.g., “Previous 3 hours”). All panels automatically adjust to reflect the selected time frame unless explicitly stated otherwise. This level of granularity enables users to analyze trends, investigate system behavior, and generate reports.

## NDTS Technical Implementation

The design goals for NDTS components deployed on spectrometer workstations were to: (1) avoid interfering with data acquisition; (2) enable simple, automatic data harvesting; (3) support diverse environments, and (4) ensure secure, reliable data transfer to the repository.

Target environments included Bruker TopSpin (v3.x and v4.x), VnmrJ, and OpenVnmrJ running on various operating system (OS) versions (RedHat, CentOS, Ubuntu, Alma, and Windows 10). These environments span ~30 years of system variability, including differences in security, system libraries, GUI support, and OS behavior.

The NDTS daemon was implemented in C++98 for maximal portability and minimal system dependency. Python was avoided due to unsupported legacy versions, and Java was excluded to reduce workstation overhead. Static linking eliminates external library dependencies, allowing deployment via a single executable. Windows support required additional effort due to POSIX non-compliance but reduced burden for users and administrators.

The daemon performs the following functions:

1. Detects when TopSpin or VnmrJ is launched, who launched it, and whether it controls the spectrometer
2. Identifies acquisition start and end events:
  **VnmrJ**: Listens to InfoProc network messages to detect transitions (e.g., from idle to acquiring)
  **TopSpin 3.x**: Uses inotify (or ReadDirectoryChanges on Windows) to monitor changes to the accounting file (enabled per user); detects experiment end
  **TopSpin 4.x**: Monitors shmem file in the user-specific TopSpin directory for both start and end times
3. Determines whether to harvest an experiment based on:
  User is configured for harvesting
  GUI harvesting toggle is “on”
  Presence of fid or ser file
  Experiment job number below a configured threshold (set by facility manager, this allows simple exclusion of “datasets” during instrument maintenance, probe testing, or other procedures by choosing an experiment number above this threshold)
  Acquisition time exceeds 1 second
  Pulse program is not topshim, rga, wobb, or gs (TopSpin only)
4. Transfers experiment directories and associated metadata (pulse program, probe info, etc.) to the NDTS gateway. If transfer fails, data is spooled locally for retry. All actions are logged with an audit trail.
5. Every 10 minutes, the daemon:
  Retries failed transfers
  Sends a heartbeat
  Requests updated user, project, study, and sample info from the gateway
  Detects if the NDTS GUI has exited and resets defaults

For manual harvesting, criteria checks that are applied on live data streams are bypassed. If an experiment was already harvested manually, it is skipped unless “Force” is selected.

The NDTS GUI, written in Tcl/Tk (also used by VnmrJ and TopSpin), maintains compatibility with versions pre- and post- Tcl 8.4. It communicates with the daemon via a dedicated TCP/IP port. Upon exit, the daemon resets user-specific settings. Multiple users can run the GUI, but settings apply only to the active spectrometer user.

The NDTS gateway is a secure, dedicated system co-located on the same network as the spectrometer workstations. Implemented in Python 3, it uses a restricted socket protocol to accept connections from authorized IPs. This protocol prevents command execution and is resilient against buffer overflows. All communication between the gateway and NAN receiver is outbound-only, relaying: heartbeats (no response expected), requests for updated metadata (checksum-based), and harvested experiment transmissions (acknowledged upon success). Failed transfers are retried; failed heartbeat/metadata requests are not. Communication occurs via HTTPS (port 443) with certificate-based authentication and short-lived access tokens that expire after 10 minutes; when a token expires, the gateway automatically obtains a new one, thwarting any attempts to reuse a stolen token. Files are transferred via SSH-based rsync (port 22) with checksums, ensuring end-to-end encryption and data integrity. Only outbound ports 443 and 22 must be open and no inbound ports are utilized. If needed, access can be restricted to the fixed IP of the NAN receiver.

The NAN receiver ingests data from gateways into a staging area. In the unlikely event that the data arrives malformed, the gateway is informed, and the data will be re-transmitted until accepted. Built in Python 3 using the Flask library, it is horizontally scalable, distributed across multiple servers. The parser service processes new experiments immediately and reprocesses missed ones every 10 minutes. It also supports batch re-parsing as metadata models evolve.

The data lifecycle is summarized:

- Staging area storage: Qumulo (https://qumulo.com) (real-time replicated). Removed once ingested into archive.
- Archive storage: Isilon (https://infohub.delltechnologies.com/en-us/l/dell-emc-smartfabric-services-with-poweredge-servers-powerstore-storage-appliance-and-isilon-storage/dell-emc-isilon-84/) for high-performance access.
- Archive disaster recovery storage: Write-once-read-many (WORM) enabled S3 bucket on HP Scality (https://www.scality.com), geo-dispersed across four data centers.
- Metadata: PostgreSQL (virtual machine based) with replication to a second server, in a separate data center, for high availability. Backups support full restoration.

This architecture ensures secure, scalable, and fault-tolerant data acquisition, transfer, and storage across multiple datacenters.

## Results

NAN is now completing its construction phase and is in the process of expanding to additional facilities, including The Ohio State University, the University of Nebraska–Lincoln, and the University of California, Santa Cruz, with further addition of NAN facility nodes on-going. While the system is still early in its deployment, it has already harvested 367,781 datasets (108,112 marked as preferred and 260,249 as redundant), encompassing 1,444 unique pulse programs from 119 registered users. To date, 570 datasets have been made public, 418 have been published with persistent identifiers, 237 knowledgebase datasets have been curated, and 2,106 datasets have been copied into NMRbox user accounts for downstream analysis. These numbers are expected to grow substantially as additional facility nodes come online.

## Discussion

The NAN Data Transport System (NDTS) demonstrates that automated harvesting and integration of NMR data across multiple facilities is both technically feasible and scalable. NDTS automatically associates immutable metadata (e.g. local user, instrument, and facility) with the dataset; allows users to attach arbitrary metadata at the time of collection (e.g. sample information and experimental parameters); and derives additional metadata (e.g. experiment dimensionality and nuclei) from parameter files generated by the instrument. Post-acquisition, the data browser provides an intuitive interface for editing mutable fields, with all changes recorded through provenance tracking to maintain integrity. The ability to re-parse existing datasets ensures that improvements or corrections to metadata can be applied retrospectively. Initial deployment has demonstrated that NDTS can reliably handle large volumes of experimental data, and with the anticipated expansion of NAN to additional facility nodes the NAN archive will become the first large-scale public repository of time-domain NMR data. For users and laboratories, this provides immediate access to their own datasets through the data browser, while the Python SDK enables efficient programmatic retrieval and the creation of curated collections suitable for machine learning and other large-scale computational studies. Network monitoring tools benefit facility managers, providing near real-time insights into local operations and facilitating report generation. Together with automated public release of datasets, the ability to publish immutable dataset versions, and robust organizational and sharing features, NDTS enhances data reuse, ensures reproducibility, and lowers barriers to discovery.

## Future Directions

Future plans for NAN include expanding the network by adding new NMR facility nodes and enhancing the cyberinfrastructure. In the second half of 2025, we anticipate that six additional U.S. NMR facilities and one international facility will join the network. Planned improvements to the NAN cyberinfrastructure include:

- Continued improvement of the portal experience for all users, including tools to reduce the administrative burden on facility managers.
- Complete the development of a Python SDK to enable programmatic access to data and metadata.
- Improve metrics monitoring for dataset downloads and views of the NAN portal pages.
- Automated analysis of pulse programs to generate metadata annotations for associated datasets.
- Automated data processing of datasets acquired using KB or other standardized pulse programs.
- Curation and export of machine learning–ready dataset collections.
- Support for multiple hubs to enhance network robustness by eliminating single points of failure and addressing data patriation laws in Europe and other regions.

## Data Availability

The NAN resource is available as a web portal (https://usnan.nmrhub.org), which provides interactive access to the data browser, sample browser, and associated management tools. All datasets originate from community users of NAN and are automatically harvested from NAN nodes and archived with rich metadata in the repository (see *Data Harvesting*). Publicly available datasets can be searched, filtered, and downloaded through the NAN data browser (see *Data and Sample Browsers*). Users without a NAN account can access all public datasets via the “Public & Knowledgebase Datasets” view located on the Resource Connector (https://usnan.nmrhub.org/resource-connector/public-datasets). Datasets become public three years after harvesting unless released earlier by the investigator, and immutable published versions are assigned persistent identifiers and are distributed under a Creative Commons Attribution (CC BY) license to support citation and reuse (see *Publishing & Public Data*). Access to embargoed non-public datasets is governed by PI controlled permissions (see *Accounts & Permissions*). In addition to the interactive web portal, NAN provides programmatic access through a Python software development kit (SDK) and RESTful API, enabling automated queries, dataset downloads, and integration into external workflows.

## Code availability

As a research infrastructure system, most NAN software has little relevance for individual investigators and therefore is not released publicly. The exception is the Python SDK, which is available on GitHub (https://github.com/NanNMR/PythonSDK) and on the Python Package Index (https://pypi.org/project/usnan/). This SDK provides programmatic access to the system. Internal components such as the NDTS spectrometer components, gateway software, receiver and parser services, along with back-end APIs are restricted for security reasons but may be made available to responsible research organizations with conditions. Organizations interested in deploying an instance of NAN cyberinfrastructure should contact the corresponding author.
